# Once-weekly teriparatide increases bone mineral density in the distal 1/10 radius, but not in the distal 1/3 radius

**DOI:** 10.1186/2193-1801-3-238

**Published:** 2014-05-08

**Authors:** Nobuo Urushibara, Naoto Kato, Ryutaro Adachi, Yasuo Nakamura, Ayumi Mihara, Toyonobu Uzawa, Shigeru Kitagawa, Masanori Hayashi, Tatsuhiko Kuroda, Teruki Sone

**Affiliations:** Department of Orthopedic Surgery, Nukada Memorial Hospital, 4-6-6 Omachi, Kamakura, Kanagawa, 248-0007 Japan; Project for Bone Metabolic Disease, Asahi Kasei Pharma Corporation, 1-105 Kanda Jinbocho, Chiyoda-ku, Tokyo, 101-8101 Japan; Hitachi Aloka Medical, Ltd, 3-7-19 Imai, Ome-shi, Tokyo, 198-8577 Japan; Department of Nuclear Medicine, Kawasaki Medical School, 577 Matsushima, Kurashiki, Okayama, 701-0192 Japan

**Keywords:** Bone mineral density, Distal radius, Dual X-ray absorptiometry, Osteoporosis, Teriparatide

## Abstract

Teriparatide significantly increases bone mineral density (BMD) of the lumbar vertebrae and femur and has a strong effect in reducing the risk of bone fractures. However, few detailed investigations with dual-energy X-ray absorptiometry (DXA) of the effects of teriparatide on the radius have been reported; specifically, there are no reports of the use of once-weekly teriparatide. In this study, the effect of once-weekly teriparatide in increasing BMD was examined in the distal 1/10 of the radius and the distal 1/3 of the radius using a DXA system for the radius. In addition, the effect of radius positioning, especially accurate correction of rotation and inclination before and after administration of teriparatide, was evaluated in an assessment of its efficacy. It was found that when positioning was corrected, a significant increase in BMD in the distal 1/10 of the radius was observed after 6 months of once-weekly teriparatide. In the distal 1/3 of the radius, no significant increase of BMD was observed. This suggests that when DXA scans of the radius are analyzed with appropriate positioning, weekly teriparatide significantly increases BMD in the distal 1/10 of the radius, which is rich in cancellous bone.

## Background

Teriparatide is a polypeptide consisting of 34 amino acids (human PTH(1-34)) from the N-terminal of human parathyroid hormone (PTH). Teriparatide promotes bone formation by increasing mature osteoblasts. This is achieved by increasing preosteoblasts through the promotion of osteoblast precursor cell differentiation and by promoting preosteoblast differentiation and inhibiting apoptosis in mature osteoblasts ([Isogai et al. 1996]) As a result, once-weekly 56.5 μg teriparatide markedly increases bone mineral density (BMD) and shows strong anti-fracture efficacy ([Nakamura et al. 2012]). Two teriparatide formulations are currently used clinically: a teriparatide 56.5 μg once-weekly formulation (weekly teriparatide) and a teriparatide 20 μg daily formulation (daily teriparatide).

Dual-energy X-ray absorptiometry (DXA) is a method of differentiating between the amounts of bone and soft tissue in the human body by irradiation with X-rays of two different energy levels, measuring the X-ray attenuation at the two respective energies, and calculating the bone mass per unit area and BMD. BMD using DXA has become the gold standard for diagnosing osteoporosis and determining treatment effectiveness because of its high level of precision and very low level of radiation exposure. Measurement sites are the lumbar vertebrae, which are rich in cancellous bone, and the femur, which is rich in cortical bone. Measurements are also possible at the distal 1/10 of the radius, which is rich in cancellous bone, and the distal 1/3 of the radius, which is rich in cortical bone.

In studies of the changes in BMD with administration of teriparatide formulations using DXA, BMD has been reported to increase significantly in the lumbar vertebrae (8.1% at 48 weeks and 13.4% at 2 years) and in the proximal femur (3.1% at 72 weeks and 3.3% at 2 years with both weekly and daily formulations ([Fujita et al. 1999];[Miyauchi et al. 2010]). With respect to the changes in BMD in the radius with teriparatide, in contrast, the only report is of a 1–2% decrease at 1 year with daily teriparatide ([Neer et al. 2001]). There are no reports of weekly teriparatide. Therefore, the changes in BMD in the radius with weekly teriparatide were investigated.

When using DXA to investigate serial changes in BMD due to drugs, it is important that the site measured is the same before and after the treatment. BMD calculated with DXA is a number obtained from image measurements, and discrepancies in the measurement site due to arm positioning at the time of the measurements are assumed to affect the measurement results. Therefore, when making accurate assessments of the efficacy of osteoporosis drugs based on BMD in a clinical study, it is important to match sites by establishing evaluation criteria for the target image data and comparing only image data that meet the evaluation criteria before and after administration of the drug. In this study, the efficacy of weekly teriparatide was evaluated after investigating the effect on BMD of changes in arm positioning using a bone phantom.

## Subjects and methods

### Subjects

The subjects were patients examined at our institution who had a vertebral compression fracture and were diagnosed with primary osteoporosis with BMD of ≤ 70% of the young adult mean (YAM) in the healthy Japanese population ([Soen et al. 2013]). The subjects of the present analysis were those who had DXA scan images of the radius taken at the start of weekly teriparatide administration and after administration for 6 months. Timing of weekly teriparatide administration was not defined such as morning or evening. Whether patients had used osteoporosis drug pretreatment or had complications affecting measurement of BMD was not considered in this study. Analysis of DXA scans of the radius was done after obtaining written, informed consent from all subjects. The study was approved by Nukada memorial hospital subcommittee.

### BMD measurement

BMD of the radius was measured using a Dichroma Scan DCS-600EXV from Hitachi Aloka Medical, Ltd. (Tokyo, Japan). The distal radius was positioned with reference to the forearm length from the styloid process of the ulna to the olecranon. The site located at 1/10 the distance of the forearm length from the styloid process of the ulna was taken as the site of the distal 1/10 radius, and the site located at 1/3 of the distance was taken as the site of the distal 1/3 radius. In the investigations of BMD in subjects, the distal radius of the non-dominant arm was taken as the measurement site.

### Effect of arm positioning

**Figure 1:**
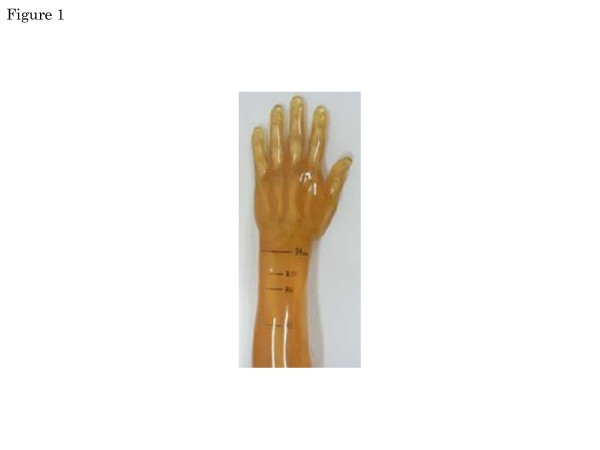
**Bone phantom.** This is Bone phantom used in this study.

When obtaining DXA scans of the radius, rotation and inclination of the arm are thought to affect BMD. Therefore, the effect of positioning changes on BMD was examined using a bone phantom. A PBU-5 whole-body phantom (Kyoto Kagaku Co., Ltd., Kyoto, Japan) was used as the bone phantom (Figure 1).

Rotation is a turning movement of the forearm by torsion of the wrist, and inclination is side displacement of the forearm by placement of the forearm at an angle with respect to the measurement area. Rotation was taken to be present when the bone phantom was rotated ±30 degrees (pronation: + direction, supination: − direction). Inclination was taken to be present when there was parallel displacement of the proximal portion of the forearm 0–7 degrees to the side. The effect on BMD of positioning changes in these respective directions was investigated. In addition to assessment of BMD at the distal 1/10 and distal 1/3 radius, the region of interest (ROI) area of the measurement site was calculated.

### Correction of BMD changes by inclination at the distal 1/10 radius

The effect of inclination can be avoided by two-dimensional rotation of the radius DXA scan image equal to the angle of inclination. This is because inclination refers to the inclination of the forearm due to placement of the forearm on an angle with respect to the measurement area. Specifically, the following manipulations were done at the distal 1/10 radius.

A line extending from the long axis of the radius within the measurement region was drawn, and this line was taken as the center line. A straight line was drawn from the styloid process of the ulna perpendicular to the measurement region, and this line was taken as the reference line. The angle obtained from the center line and reference line with the forearm placed parallel to the measurement region was taken as the reference angle. The difference between the reference angle and the angle obtained from the center line and reference line with the forearm placed obliquely to the measurement region was taken to be the inclination angle. Correction was done by rotating the image by the amount of the inclination angle.

### Assessment criteria for drug efficacy

To accurately assess drug efficacy from the radius DXA data obtained from the patients, assessment criteria that excluded the effects of rotation on BMD before and after drug administration were set. This is because it is not possible to accurately compensate for the torsion of the wrist even with two-dimensional correction of the radius DXA scan images. A percentage change in the ROI area of more than 5% was a criterion because it is thought that the drug efficacy can be detected if the change in BMD with rotation is kept within 2%, equal to a change in the ROI area of less than 5%. Therefore, patients with percentage change in the ROI area of ≥5% were excluded from the analysis.

### Statistical analysis

The data obtained with the radius DXA machine were analyzed statistically with a paired *t*-test. P values show the results of two-sided tests. SPSS statistics 20.0 (IBM Corp., Armonk, New York, NY, USA) was used for the statistical analysis.

## Results

### Effect of arm positioning on BMD measurements

The effect of arm positioning on BMD changes was investigated using a bone phantom (Figure 1). Arm positioning was evaluated with respect to the effects of two factors (rotation and inclination).

**Figure 2:**
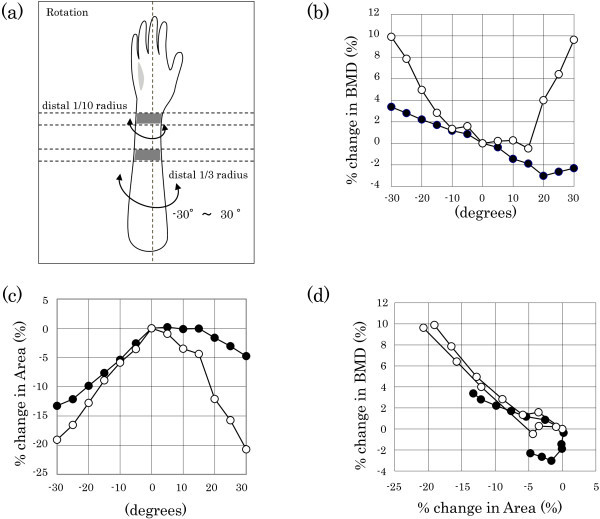
**Effect of rotation angle on changes in BMD and area.** The schema of the rotation **(a)** is shown in order to get a better understanding of the method of rotation. Changes in BMD **(b)** and area **(c)** with rotation were detected using the whole-body phantom. The relationship between BMD and area is shown **(d)**. Open circles indicate values in the distal 1/10 radius, and solid circles indicate values in the distal 1/3 radius. BMD, bone mineral density.

First, the relationships between rotation and the BMD measurement value and the ROI area were investigated. The bone phantom was rotated from -30 to 30 degrees. The schema of the rotation is shown in Figure 2a in order to get a better understanding of the method of rotation. The results of rotation revealed that, as the rotation angle became larger, BMD increased (Figure 2b), and the ROI area became smaller (Figure 2c). In addition, assessment of the correlation between the rates of change in BMD and ROI area with rotation showed a tendency for BMD to increase as the ROI became smaller (Figure 2d). Changes in BMD with rotation observed at each measurement site were a maximum of 4% for BMD and 15% for the ROI area in the distal 1/3 radius, and a maximum of 10% for BMD and 20% for the ROI area in the distal 1/10 radius. Rotation was thus shown to have a very large effect at both sites.

**Figure 3:**
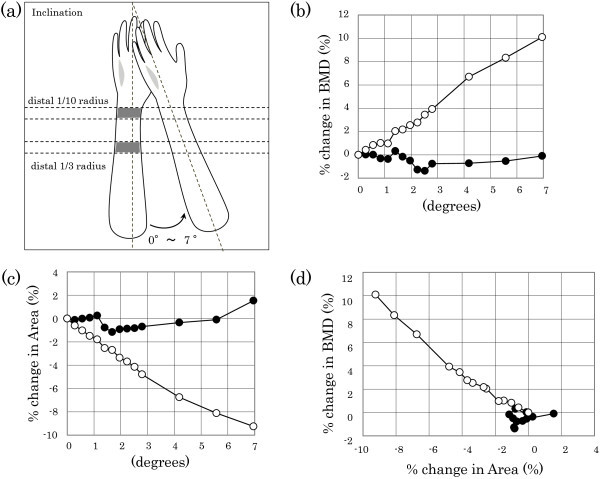
**Effect of inclination angle on changes in BMD and area.** The schema of the inclination **(a)** is shown in order to get a better understanding of the method of inclination. Changes in BMD **(b)** and area **(c)** with inclination were detected using the whole-body phantom. The relationship between BMD and area is shown **(d)**. Open circles indicate values in the distal 1/10 radius, and solid circles indicate values in the distal 1/3 radius. BMD, bone mineral density.

Next, the relationships between the inclination and BMD measurement value and the ROI area were investigated. The bone phantom was inclined from 0 to 7 degrees. The schema of the inclination is shown in Figure 3a in order to get a better understanding of the method of inclination. In the distal 1/3 radius, the inclination angle was found to have a small effect (~2%) on BMD (Figure 3b) and the ROI area (Figure 3c), and there were no correlations between inclination and BMD and the ROI area (Figure 3d). In the distal 1/10 radius, BMD increased (Figure 3b), and the ROI area became smaller (Figure 3c) as the inclination angle became larger. In addition, assessment of the relationship between the rates of change in BMD and the ROI area with inclination revealed that BMD increased as the ROI area became smaller (Figure 3d), confirming a maximum effect of 10% on BMD and the ROI area.

**Figure 4:**
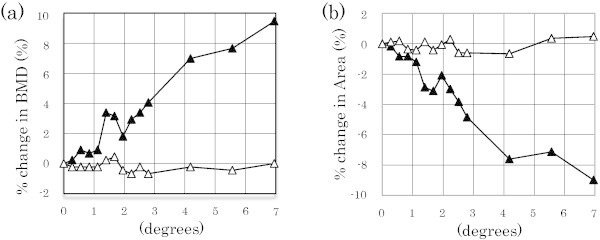
**Effect of the correction for inclination error on arm positioning.** Changes in BMD **(a)** and area **(b)** before/after correction were detected. Open triangles indicate values after correction, and solid triangles indicate values before correction in the distal 1/10 radius. BMD, bone mineral density.

Finally, the effect of correction on BMD and the ROI area was investigated. It was found that by correction with respect to the inclination, the increase in BMD (Figure 4a) and the decrease in the ROI area (Figure 4b) produced by the inclination could be reduced to a percentage change of less than 1%.

### Change of BMD by teriparatide treatment

The number of patients before correction was nine. The number of patients who fulfilled the assessment criteria after correction was five for the distal 1/10 radius and seven for the distal 1/3 radius. The patients’ characteristics are shown in Table 1. There were no significant differences in age, height, body weight, BMI, or number of prevalent vertebral fractures between all patients (before correction) and selected patients (after correction of the radius DXA scan images) (p > 0.05).

**Table 1:** **Baseline characteristics of subjects before/after corrections**

	Correction		
	Before	After	
		1/10 distal radius	1/3 distal radius
Number of patients	9	5	7
Age (years)	80.4 ± 8.3	80.2 ± 10.2	80.0 ± 8.9
Height (cm)	146.3 ± 8.0	147.0 ± 9.3	146.4 ± 9.0
Body weight (kg)	47.7 ± 7.9	50.9 ± 7.1	48.8 ± 7.1
BMI (kg/m^2^)	22.4 ± 4.4	23.9 ± 5.3	23.0 ± 4.6
Number of vertebral fractures	3.0 ± 2.4	3.6 ± 2.7	3.6 ± 2.4

BMI, body mass index.

Next, weekly teriparatide efficacy was assessed by comparing radius DXA scan images before the start of weekly teriparatide and after 6 months. When the above corrections were not made, no significant increase in BMD was found in the distal 1/10 radius (p = 0.412) or the distal 1/3 radius (p = 1.000). When corrections were made, however, a significant increase in BMD was observed in the distal 1/10 radius (p = 0.001) (Table 2). No significant increase in BMD was observed in the distal 1/3 radius (p = 0.272) (Table 3).

**Table 2:** **Changes from baseline in bone mineral density in the distal 1/10 radius before/after corrections**

	Correction			
	Before		After	
Treatment period (weeks)	0	24	0	24
Distal 1/10 radius BMD (g/cm^2^)	0.280 ± 0.076	0.284 ± 0.077	0.306 ± 0.082	0.312 ± 0.081
% increase in BMD	1.17 ± 4.21		1.98 ± 0.87	
p value (paired *t*-test)	0.412		0.001	

BMD, bone mineral density.

**Table 3:** **Changes from baseline in bone mineral density in the distal 1/3 radius before/after corrections**

	Correction			
	Before		After	
Treatment period (weeks)	0	24	0	24
Distal 1/3 radius BMD (g/cm^2^)	0.421 ± 0.051	0.421 ± 0.054	0.440 ± 0.049	0.444 ± 0.048
% increase in BMD	-0.05 ± 2.32		0.98 ± 2.17	
p value (paired *t*-test)	1.000		0.272	

BMD, bone mineral density.

## Discussion

To accurately assess the efficacy of weekly teriparatide in the distal radius, the effect of changes in arm positioning on BMD was first examined using a bone phantom. The results of the investigation of changes in BMD with inclination showed that the ROI area decreased and BMD increased with dependence on the inclination angle (Figure 1). The results of the investigation of changes in BMD with rotation, which is a three-dimensional movement, showed that, although the percentage change was limited to the distal 1/3 radius, the ROI area decreased and BMD increased at the distal 1/10 radius with dependence on the rotation angle (Figure 2).

Considering these results, it is thought to be important to define a method to avoid errors in BMD measurement due to arm positioning changes when assessing the efficacy of weekly teriparatide using a radius DXA system. The following were established as a means of minimizing arm positioning changes. 1) For inclination, radius DXA scan images were rotated two-dimensionally in the amount of the inclined angle. 2) Patients with percentage change in the ROI area of ≥5% were excluded from the analysis.

The results of the investigation of the BMD-increasing effect of weekly teriparatide in the six patients who fulfilled the assessment criteria showed a significant increase in BMD at the distal 1/10 radius (Table 2). They also showed that BMD is maintained at the distal 1/3 radius (Table 3). These results demonstrate that, under properly established conditions, radius DXA scan images show a significant increase in BMD at the distal 1/10 radius after weekly teriparatide administration for 6 months. The reason why a significant increase in BMD was observed at the distal 1/10 radius and not at the distal 1/3 radius may be explained by the difference in the volume of the cancellous bone. The content of cancellous bone is higher at the distal 1/10 radius than that at the distal 1/3 radius. Because weekly teriparatide increased cancellous bone rapidly, a significant change of BMD was observed only at the distal 1/10 radius.

The only reports on the efficacy of teriparatide for BMD of the radius are with daily teriparatide. In an investigation using DXA, Neer et al. ([2001]) reported that a 40-µg dose of teriparatide decreased bone mineral density at the shaft of the radius by two more percentage points. Michalska et al. ([2012]) investigated the efficacy of daily teriparatide at the distal 1/3 radius using DXA and reported a significant decrease in BMD of 2–3% at 6 months. Using the results of hr-pQCT, Macdonald et al. ([2011]) reported that BMD of the distal radius decreased with daily teriparatide. Based on these results, one could conclude that BMD of the radius decreases with daily teriparatide.

There is a difference in the direction of the changes in BMD between these previous findings and the present results. The detailed mechanism for this difference is unclear, but it may be explained in part by metabolic bone markers. The major difference between weekly teriparatide and daily teriparatide is the dynamics of the bone resorption marker urine cross-linked N-telopeptide of type I collagen (NTX). With teriparatide administration for 6 months, urine NTX showed a percentage change of -5% from the time administration was started in the case of weekly teriparatide ([Nakamura et al. 2012]), but with daily teriparatide, the percentage change was +50% ([McClung et al. 2005]). Thus, daily teriparatide may have caused a decrease in the BMD of radius sites as a result of strongly renewing bone resorption. This is similar to a report stating that daily teriparatide increases the porosity of the cortical bone of the radius using hr-pQCT ([Macdonald et al. 2011]). Weekly teriparatide, in contrast, is thought to increase BMD at the distal 1/10 radius and maintain the BMD at the distal 1/3 radius because it inhibits bone resorption.

In any case, there is a limitation that bone metabolic markers were not measured in this study. Further clinical studies are needed to evaluate the relationship between change in bone metabolic markers and BMD. To support the above, a bone structural analysis using hr-pQCT in patients administered weekly teriparatide may elucidate the details of the effect of weekly teriparatide in the radius. Such a study is also needed.

## Conclusions

A method to avoid irrelevant measurements of BMD produced by inadequate arm positioning when measuring BMD in the radius was developed, and it was demonstrated that BMD at the distal 1/10 radius was significantly increased and BMD at the distal 1/3 radius was maintained after 6 months of once-weekly teriparatide treatment. However, further studies with more participants are needed to provide clear evidence of the pharmaceutical effect of once-weekly teriparatide using the method established in this paper.
